# Green PEGylation of Graphene Oxide via Diels–Alder Chemistry in Deep Eutectic Solvents for pH‐Responsive Doxorubicin Delivery

**DOI:** 10.1155/tswj/9094354

**Published:** 2026-08-04

**Authors:** Thi Nhat Thang Nguyen, Thao Quynh Ngan Tran, Xuan Thang Cao

**Affiliations:** ^1^ Faculty of Chemical Engineering, Industrial University of Ho Chi Minh City, Ho Chi Minh City, Vietnam, hui.edu.vn

**Keywords:** deep eutectic solvents, Diels–Alder, doxorubicin, drug delivery, graphene oxide, PEGylation

## Abstract

Graphene oxide (GO), a two‐dimensional carbon nanomaterial, exhibits a large surface area, rich oxygen‐containing functionalities, and favorable aqueous dispersibility, making it a suitable platform for drug delivery. However, traditional covalent modification strategies frequently involve multistep procedures or the use of hazardous organic solvents. In this study, GO was functionalized via a novel green strategy: Diels–Alder (DA) cycloaddition of maleic anhydride (MA) utilizing deep eutectic solvents (DESs) as a sustainable reaction medium. The resulting GO‐PEG nanocarriers, formed through subsequent esterification with poly(ethylene glycol) lithium alkoxide, were thoroughly characterized by FTIR, Raman spectroscopy, XRD, and TGA. This DES‐based approach distinguishes itself from prior DA functionalizations by offering an environmentally benign pathway that maintains high grafting efficiency without the need for toxic volatile media. The optimized DOX/GO‐PEG nanohybrids exhibited a high doxorubicin (DOX) loading capacity of 128.4 wt.% at a feed ratio of 150:1 (DOX/GO‐PEG, w/w) and demonstrated pH‐responsive drug release—77.3% at pH 5.0 and 36.3% at pH 7.4 after 72 h. Cytotoxicity studies showed that GO‐PEG exhibited negligible toxicity to HEK293 cells, whereas DOX/GO‐PEG induced potent, dose‐dependent cytotoxicity in HeLa cancer cells. Confocal imaging confirmed DOX nuclear accumulation in treated HeLa cells. This study provides a first‐time demonstration of DES‐mediated DA PEGylation on GO, establishing a high‐efficiency and eco‐friendly framework for advanced nanocarrier synthesis.

## 1. Introduction

Graphene oxide (GO) has gained widespread interest as a multifunctional platform for drug delivery due to its two‐dimensional sheet structure, large surface area, and abundance of oxygen‐containing functional groups such as hydroxyl, carboxyl, and epoxy moieties, which facilitate both physical interactions and chemical modifications with therapeutic agents [1–5]. These groups enable *π*–*π* stacking, hydrogen bonding, and electrostatic interactions with drugs like doxorubicin (DOX), allowing for efficient drug loading and pH‐responsive release [6–8]. Compared with one‐dimensional carbon nanotubes (CNTs), GO exhibits superior aqueous dispersibility, better surface accessibility, and improved safety profiles, making it a more favorable candidate for biomedical applications [9, 10]. In addition to its physicochemical versatility, GO‐based nanocarriers have demonstrated significant promise in tumor‐targeted delivery, multimodal imaging, and combinatorial therapies, contributing to the advancement of cancer nanomedicine [11–13].

Despite these advantages, unmodified GO can induce oxidative stress, potential cytotoxicity, and aggregation in physiological environments, which limits its direct use in vivo [14–16]. To improve biocompatibility, stability, and circulation time, surface functionalization approaches, especially PEGylation, have been widely applied [17, 18]. PEGylation not only enhances aqueous solubility and reduces immunogenicity but also prolongs systemic circulation, which is critical for achieving effective drug accumulation at tumor sites. Recent developments include the use of linear and branched PEG derivatives, offering improved stealth behavior and drug conjugation flexibility [19–21]. While effective, most conventional PEGylation strategies rely on multistep processes involving chloroacetic acid for carboxylation and specialized coupling reagents, such as N‐ethyl‐N ^′^‐(3‐dimethylamino‐propyl) carbodi‐imide hydrochloride (EDC) and N‐hydroxysuccinimide (NHS), to facilitate covalent grafting. These methods often require meticulous pH control and the use of excess chemical additives, which can lead to limited grafting efficiency or unintended structural degradation of the GO lattice [16–20]. Transitioning these processes to a more sustainable framework remains a challenge in nanomaterial engineering.

In contrast, Diels–Alder (DA) chemistry offers a mild, catalyst‐free, and orthogonal strategy for functionalizing carbon materials [21–23]. This reaction proceeds under ambient or moderately elevated temperatures and is compatible with a wide range of functional groups, enabling controlled covalent modification without compromising the core structure. When combined with green solvents such as deep eutectic solvents (DESs), which are biodegradable, tunable, and nontoxic, DA chemistry becomes an attractive platform for sustainable nanomaterial design [24–26]. Specifically, the use of DES as a reaction medium offers significant advantages over traditional organic solvents by providing a high‐polarity environment that can stabilize the DA transition state, thereby enhancing reaction efficiency under environmentally benign conditions. This strategy aligns with key green chemistry principles, specifically achieving high atom economy through the addition‐based DA mechanism and reducing the environmental factor (E‐factor) by replacing volatile organic solvents with biodegradable DES. Although DA reactions in DESs have been applied to CNTs [27, 28], very few studies have explored this strategy for GO functionalization, representing a significant gap in the literature [29]. Developing a DA‐based, DES‐mediated PEGylation approach for GO not only addresses these limitations but also aligns with current trends in scalable and eco‐friendly drug delivery system design.

In this study, we developed a novel DA‐based strategy for grafting MA onto GO sheets in a DES composed of choline chloride (ChCl) and ethylene glycol (EG), followed by PEGylation through esterification with PEG lithium alkoxide. This dual‐step functionalization approach enables the fabrication of biocompatible and pH‐sensitive GO‐PEG nanocarriers for DOX delivery. The system was characterized by using a suite of spectroscopic and microscopic techniques, and its cytotoxicity, drug loading capacity (DLC), and release behavior were systematically evaluated. The synthetic route is illustrated in Scheme 1 and includes green DA functionalization and PEGylation, followed by DOX loading and biological evaluation.

**Scheme 1:**
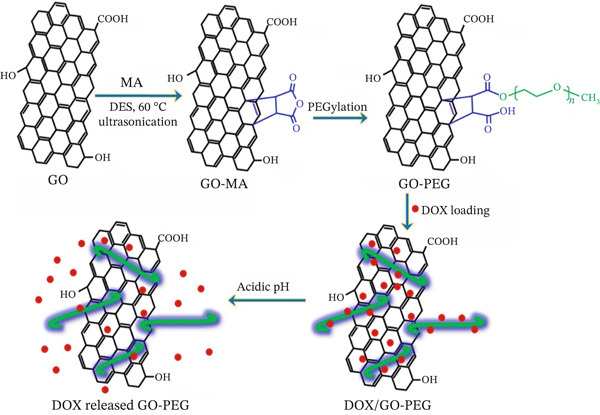
Synthetic pathway for the preparation of GO‐PEG and DOX/GO‐PEG nanocarrier via DA reaction and esterification.

## 2. Experimental Details

### 2.1. Materials

GO was purchased from Graphenea (Spain). Maleic anhydride (MA), ChCl, EG, tetrahydrofuran (THF), poly(ethylene glycol) methyl ether (Mn = 4600 g/mol), and other chemicals were purchased from Sigma‐Aldrich (United States) and used without further purification. Doxorubicin hydrochloride (DOX·HCl) was provided by Boryung Pharm. Co. (Korea).

### 2.2. Functionalization of GO via DA and PEGylation

GO (50 mg) was dispersed in 30 mL of a DES composed of ChCl and EG (molar ratio 1:2) and ultrasonicated for 30 min. ChCl:EG was selected as the reaction medium due to its low viscosity at the operating temperature (60°C) and excellent capacity to stabilize GO dispersions, ensuring maximum surface exposure for the DA cycloaddition. MA (200 mg) was added, and the mixture was stirred at 60°C for 6 h to allow DA functionalization. The product (GO‐MA) was collected by centrifugation, washed thoroughly with ethanol and deionized water, and vacuum‐dried (mass yield: 73.4% based on the initial mass of GO).

PEG lithium alkoxide was prepared by reacting 15 mL of PEG solution in THF with 3.6 mL of n‐butyllithium (1.6 M in hexane) under nitrogen atmosphere for 30 min (handled strictly under anhydrous and inert conditions for safety). GO‐MA was redispersed in dry THF and mixed with PEG lithium alkoxide at room temperature for 24 h. The PEGylated GO (GO‐PEG) was purified by repeated centrifugation and washing with THF and water (mass yield: 77.2% based on the mass of GO‐MA).

### 2.3. DOX Loading and In Vitro Release

GO‐PEG (100 *μ*g/mL in PBS, pH 8.0) was incubated with DOX solution (1000 *μ*g/mL in PBS, pH 8.0) at varying feed ratios (w/w = 0.1–2) under dark conditions for 24 h. The loaded hybrids (DOX/GO‐PEG) were separated by filtration, and the unbound DOX in the filtrate was quantified by UV‐Vis spectroscopy at 490 nm. The DLC and drug loading efficiency (DLE) were calculated by using the following equation: DLC(*%*)  =  (weight of loaded DOX/weight of GO  −  PEG nanocarrier) × 100 and DLE(*%*)  =  (weight of loaded DOX/weight of DOX in feed) × 100. Drug release studies were performed at pH 7.4, pH 6.5, and pH 5.0 (PBS, 37°C) using a dialysis bag (MWCO = 13 kDa). At predetermined intervals (2–72 h), aliquots were collected and analyzed for DOX content.

### 2.4. Cell Culture and Cytotoxicity

HEK293 (normal human embryonic kidney) and HeLa (cervical cancer) cell lines were cultured in DMEM with 10% FBS and 1% penicillin/streptomycin at 37°C, 5% CO_2_. Cell viability was measured using WST‐1 assay. Cells were seeded in 96‐well plates (1 × 10^4^/well) and incubated for 24 h. HEK293 cells were treated with GO‐PEG (0–100 *μ*g/mL), and HeLa cells were treated with DOX or DOX/GO‐PEG (0.1–15 *μ*g/mL DOX equivalent) for 24 or 48 h. WST‐1 solution (10 *μ*L) was added, incubated for 3 h, and absorbance was measured at 460 nm.

### 2.5. Confocal Microscopy

HeLa cells were seeded onto glass‐bottom dishes and treated with 15 *μ*g/mL DOX or DOX/GO‐PEG (DOX equivalent) for 24 h. Cells were fixed and stained with DAPI (1 *μ*g/mL), and nuclear morphology was imaged by confocal laser scanning microscopy (CLSM, Zeiss LSM 710).

### 2.6. Characterization

The structures of GO, GO‐MA, and GO‐PEG were analyzed by FTIR spectroscopy (KBr pellet), Raman spectroscopy (JASCO NRS‐5000, 532 nm laser), XRD (Cu K*α*), and TGA (PerkinElmer Pyris 1, 50°C–700°C, 10°C/min). Morphology was observed by SEM. DOX concentration was measured by UV‐Vis spectrophotometry.

### 2.7. Statistical Analysis

All experimental data, including drug release and cell viability assays, were performed in triplicate (*n* = 3) and expressed as mean ± standard deviation. Statistical significance was evaluated using one‐way ANOVA followed by Tukey′s post hoc test, with a *p* value of < 0.05 considered statistically significant.

## 3. Result and Discussion

### 3.1. Synthesis of GO‐PEG

FTIR spectra of pure GO, GO‐MA, and GO‐PEG are presented in Figure 1a. The pure GO displayed characteristic absorption bands at approximately 3410 cm^−1^ (O–H stretching), 1726 cm^−1^ (C=O stretching from carboxylic acid groups), 1620 cm^−1^ (C=C skeletal vibration from unoxidized graphitic domains), 1384 cm^−1^ (C–OH bending), and 1056 cm^−1^ (C–O stretching from epoxy and alkoxy groups), consistent with previous reports. After DA reaction with MA, the FTIR spectrum of GO‐MA showed new peaks at 1577 and 1475 cm^−1^, which are attributed to the skeletal vibration of the newly formed six‐membered ring and bending vibrations of C–H groups introduced via MA, respectively. The bands at 1716 and 1620 cm^−1^, also present in pure GO, were retained, likely reflecting overlapping signals from C=O stretching and residual aromatic domains. Following PEGylation, GO‐PEG displayed additional peaks at 2852–2920 cm^-1^ (–CH_2_– symmetric and asymmetric stretching), confirming the successful grafting of PEG chains onto the GO‐MA surface. These changes in the FTIR spectra confirm the chemical modification of GO via DA reaction and esterification. The plausibility of the DA reaction is supported by the unique environment of the ChCl:EG medium. Beyond its role as a green solvent, the DES acts as a multifunctional medium that provides both catalytic and stabilizing effects. Specifically, the extensive hydrogen‐bonding network between the EG/choline components and the carbonyl groups of MA acts as a mild Bronsted acid catalyst, polarizing the dienophile and lowering the activation energy for the [4 + 2] cycloaddition. Simultaneously, the structured DES environment stabilizes the transition state, allowing the reaction to proceed efficiently at a moderate temperature of 60°C while preserving the structural integrity of the GO basal plane.

**Figure 1:**
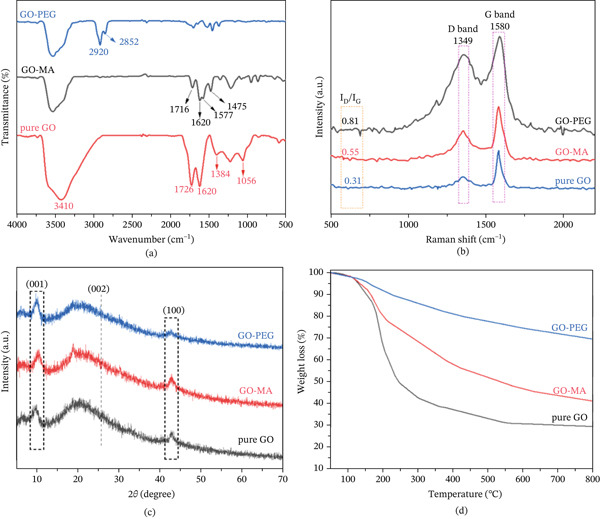
(a) FT‐IR spectra, (b) Raman spectra, (c) XRD patterns, (d) and TGA curves of pure GO, GO‐MA, and GO‐PEG.

Raman spectra of pure GO, GO‐MA, and GO‐PEG are shown in Figure 1b. The pristine GO exhibited two prominent bands at approximately 1349 cm^−1^ (D band) and 1580 cm^−1^ (G band), corresponding to the disorder‐induced A1g breathing mode and the in‐plane E2g vibration of sp^2^ carbon atoms, respectively. The intensity ratio of the D–G band (I_D_/I_G_) for pure GO was measured to be 0.31, indicative of a moderate level of structural defects and oxidized domains. Upon DA functionalization with MA, the I_D_/I_G_ ratio increased to 0.55 in GO‐MA, reflecting the introduction of additional defects and disruption of the sp^2^ carbon network due to covalent grafting. After PEGylation, the I_D_/I_G_ ratio further increased to 0.81 in GO‐PEG, suggesting further functionalization and transformation of sp^2^‐hybridized domains to sp^3^ hybridized carbon structures. This progressive increase in I_D_/I_G_ ratio confirms the stepwise chemical modification of GO and the successful surface engineering of the nanocarrier. It is worth noting that this trend also reflects the approach toward a disordering limit, where the increase in the D band intensity is not only due to sp^2^–sp^3^ conversion but also accounts for the reduction in the average size of the sp^2^ clusters and the formation of new edge defects during the functionalization process. This dual contribution provides a more comprehensive understanding of the structural evolution from pristine GO to the highly functionalized GO‐PEG system.

XRD patterns of pure GO, GO‐MA, and GO‐PEG are presented in Figure 1c. Pure GO exhibited a sharp peak at 2*θ* ≈ 10.2°, corresponding to the (001) plane, which is characteristic of layered GO structures with an interlayer spacing of approximately 0.87 nm. A broad peak around 2*θ* ≈ 42 –44° was also observed, attributed to the (100) reflection from in‐plane periodicity within the graphene sheets. Upon functionalization with MA and PEG, the (100) reflection became broader and less intense, further supporting the successful surface modification and reduced crystallinity. The absence of a well‐defined (002) peak near 2*θ* ≈ 26° across all samples confirms that the materials remained predominantly amorphous with disordered stacking after functionalization.

TGA analysis was conducted to assess the thermal stability and degree of functionalization of the GO‐based materials (Figure 1d). Pure GO exhibited a major weight loss around 120°C–250°C due to the decomposition of labile oxygen‐containing functional groups, such as carboxylic acids, hydroxyls, and epoxides. A second gradual weight loss was observed up to 560°C, attributed to pyrolysis of the carbon framework. In contrast, GO‐MA showed increased thermal stability with additional weight loss steps occurring between 200°C and 350°C, which are associated with the decomposition of MA moieties covalently attached to the GO surface. The total weight loss was higher than that of pure GO, confirming successful MA grafting. The TGA curve of GO‐PEG displayed a further shift in thermal decomposition with a major mass loss above 300°C, corresponding to the degradation of PEG chains. The overall weight loss of GO‐PEG was significantly higher compared with GO and GO‐MA, indicating the presence of a substantial amount of grafted polymer. These results provide further evidence of stepwise surface modification and improved thermal stability of the PEGylated GO nanocarrier.

### 3.2. Drug Loading and In Vitro Drug Release

To evaluate the drug‐carrying potential of the PEGylated GO nanocarriers, DOX was loaded onto GO‐PEG at various feed ratios ranging from 10 to 200 (w/w, DOX to GO‐PEG). The DLC and DLE are summarized in Table 1. A progressive increase in DLC was observed with increasing feed ratios, reaching a maximum of 128.4 wt.% at a feed ratio of 150. At this point, DLE also peaked at 78.7%. However, further increase of the feed ratio to 200 resulted in a decline in DLC, which could be attributed to saturation of available adsorption sites and partial drug aggregation or desorption during washing. Based on these results, the feed ratio of 150 was selected as the optimal condition for subsequent experiments. The high DLC of GO‐PEG can be attributed to multiple interaction mechanisms between the DOX molecules and the GO surface, including *π*–*π* stacking with the sp^2^‐hybridized carbon domains, hydrogen bonding with surface hydroxyl and carboxyl groups, and electrostatic interactions. Furthermore, PEGylation enhances the hydrophilicity and dispersibility of the carrier, allowing greater exposure of active binding sites. Notably, the maximum DLC achieved in this study (128.4 wt.%) is substantially higher than many previously reported PEGylated GO systems, which typically exhibit loading capacities below 100 wt.% [19, 30]. This superior performance can be attributed to the DA functionalization in DES, which, unlike aggressive chemical oxidation or conventional covalent grafting, effectively preserves the aromatic *π*‐conjugation of the GO basal plane. Consequently, it maintains a larger hydrophobic surface area for *π*–*π* stacking with DOX molecules [31]. Similar trends of high loading capacity have been observed in other green‐functionalized carbon systems, confirming the advantages of maintaining structural integrity for drug delivery applications.

**Table 1:** Summary of DLC and DLE at various DOX to GO‐PEG feed ratios.

No.	Feed ratio (w/w) m_DOX_/m_GO-PEG_	DLC (wt.%)	DLE (wt.%)
1	10	37.4 ± 3.2	53.1 ± 2.2
2	50	46.1 ± 3.4	65.2 ± 2.3
3	100	113.3 ± 3.1	69.3 ± 3.1
4	150	128.4 ± 3.6	78.7 ± 3.3
5	200	104.3 ± 3.4	71.3 ± 3.0

In vitro drug release profiles were examined under different pH conditions (pH 7.4, pH 6.5, and pH 5.0) to simulate physiological and endosomal/tumoral environments, respectively. The DOX/GO‐PEG hybrids were incubated in PBS at 37°C, and the cumulative drug release was monitored over 72 h. As shown in Figure 2, a pronounced pH‐responsive release behavior was observed. At pH 7.4 and pH 6.5, only 36.3% and 57.8% of the loaded DOX was released after 72 h, whereas at pH 5.0, the cumulative release reached approximately 77.3%. This accelerated release at acidic pH can be explained by the increased solubility of DOX under acidic conditions and the weakening of hydrogen bonding and electrostatic interactions, which facilitates drug diffusion from the carrier. Such pH‐sensitive behavior is desirable for tumor‐targeted drug delivery, as it minimizes drug leakage under normal physiological conditions while enabling controlled release in the acidic tumor microenvironment. Together, these findings demonstrate that GO‐PEG not only enables high drug payloads but also provides a responsive release mechanism, making it a promising platform for smart drug delivery systems in cancer therapy.

**Figure 2:**
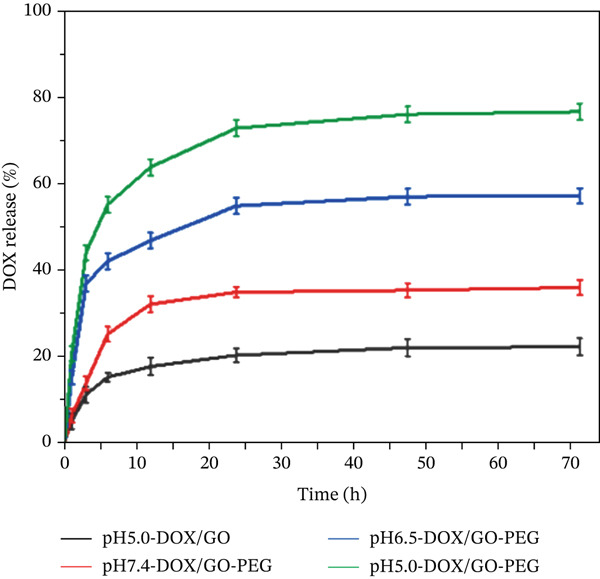
pH‐responsive release profile of DOX from DOX/GO‐PEG at different pH conditions.

### 3.3. Cytotoxicity Evaluation of GO‐PEG and DOX/GO‐PEG

The biocompatibility of GO‐PEG and the therapeutic efficacy of DOX/GO‐PEG were assessed using WST‐1 assays on HEK293 (normal human embryonic kidney) and HeLa (cervical cancer) cell lines, respectively. GO‐PEG was incubated with HEK293 cells at various concentrations ranging from 20 to 100 *μ*g/mL for 24 h. As shown in Figure 3, GO‐PEG exhibited negligible cytotoxicity up to 100 *μ*g/mL, maintaining over 95% cell viability, indicating that the PEGylated GO nanocarrier is largely biocompatible and safe for use in normal cells. In contrast, the cytotoxicity of DOX/GO‐PEG was evaluated on HeLa cells at DOX‐equivalent concentrations of 0.1–15 *μ*g/mL, with incubation times of 24 h (Figure 3). A dose‐dependent decrease in cell viability was observed, with values dropping to 44.1% and 38.8% after 24 h of treatment at 10 and 15 *μ*g/mL, respectively, indicating increasing cytotoxicity with higher drug concentrations. The free DOX group showed higher cytotoxicity in early time points, consistent with its faster cellular uptake, whereas DOX/GO‐PEG exhibited a delayed but sustained cytotoxic effect, attributable to the controlled release of DOX from the nanocarrier. These results suggest that GO‐PEG itself is nontoxic to normal cells, whereas DOX/GO‐PEG retains potent anticancer activity. The slower onset of cytotoxicity in the nanocarrier‐treated group may provide an advantage by reducing burst toxicity and allowing for sustained therapeutic effects in tumor environments. Together, these findings highlight the potential of DOX/GO‐PEG as a safe and effective drug delivery system for cancer therapy.

**Figure 3:**
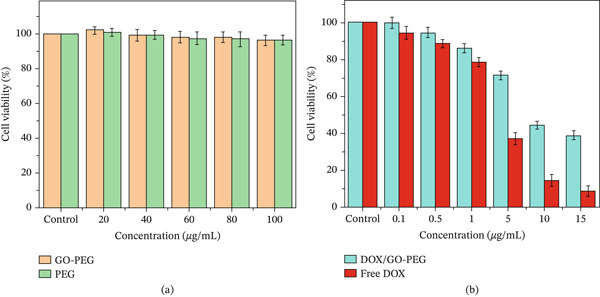
(a) HEK293 cell viability obtained by WST‐1 method after 24 h incubation with different concentrations of GO‐PEG, and (b) HeLa cell viability obtained by WST‐1 method after 24 h incubation with different concentrations of DOX/GO‐PEG and free DOX. The error bars above the bars indicate ±standard deviation (*n* = 3).

### 3.4. Cellular Uptake and Apoptosis Induction of DOX/GO‐PEG

To investigate the intracellular distribution of DOX and the mechanism of anticancer activity, CLSM was performed on HeLa cells treated with free DOX and DOX/GO‐PEG for 24 h at an equivalent DOX concentration of 15 *μ*g/mL. DAPI staining was used to visualize cell nuclei (blue), whereas the intrinsic fluorescence of DOX (red) enabled monitoring of drug localization (Figure 4). In untreated control cells, nuclei appeared intact and uniformly stained with DAPI, showing no signs of damage. Cells treated with free DOX showed strong red fluorescence primarily localized within the nucleus, indicating rapid cellular uptake and direct nuclear accumulation of the drug. In contrast, DOX/GO‐PEG–treated cells displayed relatively weaker but gradually increasing red fluorescence over time, initially distributed in the cytoplasm and later accumulating in the nucleus. This observation suggests a delayed yet effective internalization of DOX through controlled release from the GO‐PEG carrier. Additionally, nuclear condensation and fragmentation were observed in DOX/GO‐PEG–treated cells, indicative of apoptosis induction. These morphological changes are closely linked to the specific apoptotic pathways triggered by DOX. Once released from the GO‐PEG carrier and translocated into the nucleus, DOX intercalates into the DNA and inhibits topoisomerase II, leading to irreversible DNA double‐strand breaks and subsequent nuclear damage. Furthermore, the localized intracellular delivery of DOX via the nanoplatform may enhance the generation of reactive oxygen species (ROS), which further promotes mitochondrial dysfunction and exacerbates the observed nuclear degradation. Compared with free DOX, which enters cells quickly and initiates early cytotoxic effects, the GO‐PEG nanocarrier facilitates a more sustained delivery and apoptotic response, potentially enhancing therapeutic selectivity. These imaging results corroborate the cytotoxicity assay findings and confirm that DOX/GO‐PEG not only enters cancer cells but also delivers the drug effectively to the nucleus, thereby inducing programmed cell death. This supports the role of PEGylated GO as a viable and responsive platform for intracellular delivery of chemotherapeutic agents. Furthermore, the practical viability of the developed GO‐functionalization strategy was evaluated in terms of scalability and recyclability. The synthesis of ChCl:EG DES is an atom‐economical process that can be easily scaled up without the need for complex purification or specialized infrastructure, representing a significant advantage for industrial‐scale nanomedicine production. Importantly, the DES medium can be recovered through vacuum filtration and simple dehydration, showing high potential for reuse in subsequent DA cycles. This recyclability not only reduces the cost of production but also strictly adheres to green chemistry principles by minimizing hazardous chemical waste. These features, combined with the superior DLC and biocompatibility, underscore the robustness of the DES‐mediated DA approach for the fabrication of advanced drug delivery systems with high biomedical and industrial relevance.

**Figure 4:**
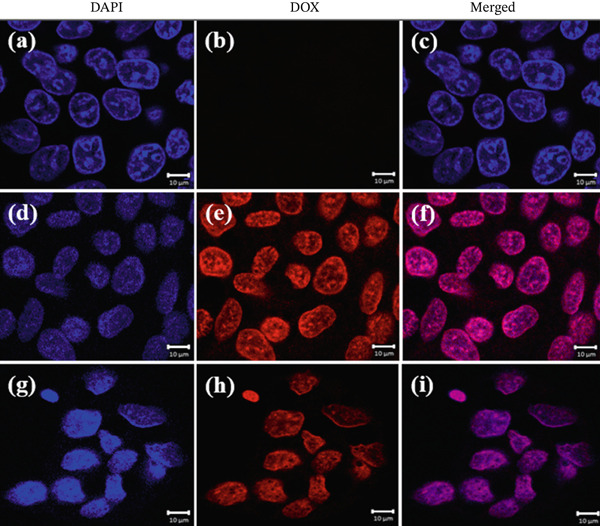
Confocal laser scanning microscopy images of HeLa cells after 24 h incubation with free DOX and DOX/GO‐PEG (DOX equivalent: 15 *μ*g/mL). Each panel presents images from left to right: DAPI‐stained cell nuclei (blue), DOX fluorescence (red), and the merged image. (a–c) Untreated control cells, (d–f) cells treated with DOX/GO‐PEG, and (g–i) cells treated with free DOX, scale bar = 10 *μ*m.

## 4. Conclusion

In this study, we developed a novel and sustainable GO‐based nanocarrier for pH‐responsive DOX delivery through a two‐step functionalization process: DA grafting of MA in DES, followed by esterification with PEG lithium alkoxide. Structural analyses via FTIR, Raman spectroscopy, XRD, and TGA confirmed the successful and progressive surface modification of GO. The resulting GO‐PEG nanocarriers exhibited excellent drug‐loading performance, with a maximum loading capacity of 128.4 wt.% at a DOX: GO‐PEG feed ratio of 150. In vitro release studies revealed pronounced pH‐sensitive behavior, with significantly higher DOX release at pH 5.0 (77.3%) compared with pH 7.4 (36.3%) after 72 h. Cytotoxicity assays confirmed that GO‐PEG was nontoxic to normal HEK293 cells, whereas DOX/GO‐PEG effectively induced dose‐dependent cytotoxicity in HeLa cancer cells. Confocal imaging further demonstrated intracellular DOX delivery and nuclear accumulation, accompanied by apoptosis‐related nuclear fragmentation. These findings demonstrate that the GO‐PEG nanocarrier serves as a biocompatible, high‐capacity, and pH‐responsive platform, where the use of DES‐mediated DA chemistry provides a more sustainable and efficient alternative to traditional functionalization methods. This approach effectively addresses current limitations by eliminating toxic coupling agents and minimizing structural damage to the GO lattice, which in turn facilitates superior drug loading performance. The high stability and safety of this system, achieved through these milder synthesis conditions, pave the way for future in vivo investigations to evaluate its systemic biodistribution and therapeutic potential in complex biological environments. Moreover, the inherent versatility of this green DA‐functionalization strategy offers a robust framework that can be readily adapted for the surface engineering of other carbon‐based nanomaterials, broadening the horizons of sustainable nanotechnology in targeted cancer therapy.

## Funding

No funding was received for this manuscript.

## Conflicts of Interest

The authors declare no conflicts of interest.

## Data Availability

The data that support the findings of this study are available from the corresponding author upon reasonable request.
